# Reducing the risk of non-sterility of aseptic handling in hospital pharmacies, part C: applying risk assessment and risk control in practice

**DOI:** 10.1136/ejhpharm-2021-002747

**Published:** 2021-07-07

**Authors:** Frits A Boom, Paul P H Le Brun, Judith M Ris, Tjitske Veenbaas, Daan Touw

**Affiliations:** 1 Department of Clinical Pharmacy, Zaans Medical Centre, Zaandam, The Netherlands; 2 Department of Clinical Pharmacy and Pharmacology, University Medical Centre Groningen, Groningen, The Netherlands; 3 Department of Clinical Pharmacy & Toxicology, Leiden University Medical Center, Leiden, The Netherlands; 4 Department of Clinical Pharmacy, Albert Schweitzer Ziekenhuis, Dordrecht, The Netherlands

**Keywords:** aseptic preparation, audit, pharmaceutical preparations, pharmacopoeia, pharmacy service, hospital

## Abstract

**Objectives:**

To describe the application of the model described in part A and part B of this series of articles for risk assessment (RA) and risk control (RC) of non-sterility during aseptic handling. The model was applied in nine hospital pharmacies.

**Methods:**

The starting point was an audit of each hospital pharmacy. The determined risk reduction and remaining risks were entered into a risk assessment model. The corresponding risk prioritisation numbers (RPNs) for each source of risk were calculated and these values were summed up to a cumulative RPN. Subsequently, all hospital pharmacies started an improvement programme, using the risk assessment as input. Results of aseptic process simulation (APS) and microbiological monitoring (MM) were also collected. The participants were informed about their progress of risk reduction and results of APS and MM during the study period. At the end of the study (about 4 years after the start), a final assessment was executed by using a checklist with risk reducing measures for each source of risk. Additional risk reduction and remaining risks were put in an RA and RC template and corresponding RPN values and a new cumulative RPN were determined.

**Results:**

At the start of the study differences in cumulative RPN values were relatively small (from 630 to 825). At the end they were relatively great (from 230 to 725), which illustrates a different sense of urgency for reducing the risk of non-sterility. Of all the risk reducing measures, a yearly audit of all operators had the greatest impact on reducing the risk of non-sterility. Except for glove prints, there was no correlation between process improvement (lower cumulative RPN) and results of microbiological controls.

**Conclusion:**

A systematic and science-based reduction of the risks of non-sterility can be done by using a checklist with risk reducing measures and an RA & RC template. Prospectively, the relevance of each risk reducing measure can be demonstrated by RPN calculations. Microbiological controls are an important part of the overall assurance of product quality. However, the results are less useful for assessing the risk of non-sterility.

## Introduction

Aseptic handling is the procedure to enable sterile products to be made ready to administer using closed systems.^1^ Because of the risk of medication errors and the chance of microbiological contamination during preparation, aseptic handling is recognised as a high-risk procedure.^1–3^

In part A and part B of this series of articles we described a model for risk assessment (RA) and risk control (RC) of non-sterility during aseptic handling.^4 5^ Risk reducing measures, for each source of risk, were listed and remaining risks were quantified by using risk prioritisation numbers (RPNs). Nearly all sources of risk could be reduced to a safe level. However, touching critical spots as well as remaining micro-organisms after disinfection on stoppers or ampoule necks will still give a small risk of non-sterility. Besides, if aseptic handling is executed in a safety cabinet, the risk of blocking first air on critical spots cannot be completely excluded (‘first air’ and ‘critical spot’: definitions are given in online supplemental file 1).

10.1136/ejhpharm-2021-002747.supp1 Supplementary data

The application of the developed RA and RC model and the effect of reducing the risk of non-sterility during aseptic handling in nine hospital pharmacies is described in this article. Implementation of risk reducing measures for each source of risk after an initial audit were tracked during a period of 4 years and evaluated after a final assessment. Results were expressed as a reduction of the RPN values.

In addition, if the chance of non-sterility has been reduced, better results of microbiological controls such as aseptic process simulation (APS) and microbiological monitoring (MM) are likely to be expected. Therefore, APS and MM were assessed as secondary outcomes.

The study focuses on non-hazardous products. However, most of the results and recommendations are also applicable to aseptic handling of hazardous products. There is only little experience with isolators in the Netherlands. Therefore, as in the previously published parts A and B, we restricted this study to aseptic handling done in a laminar airflow cabinet (LAF) or safety cabinet (SC).^4 5^

## Materials and methods

### Participating hospital pharmacies

Nine different kinds of hospital pharmacies (regional, top clinical and university) participated in this study. In these hospital pharmacies aseptic handling was carried out by well trained pharmaceutical technicians. Procedures are according to the chapter ‘Aseptic handling of the Dutch GMP-hospital pharmacy’.^6^

### Assessing aseptic handling in nine hospital pharmacies

**Figure 1:**
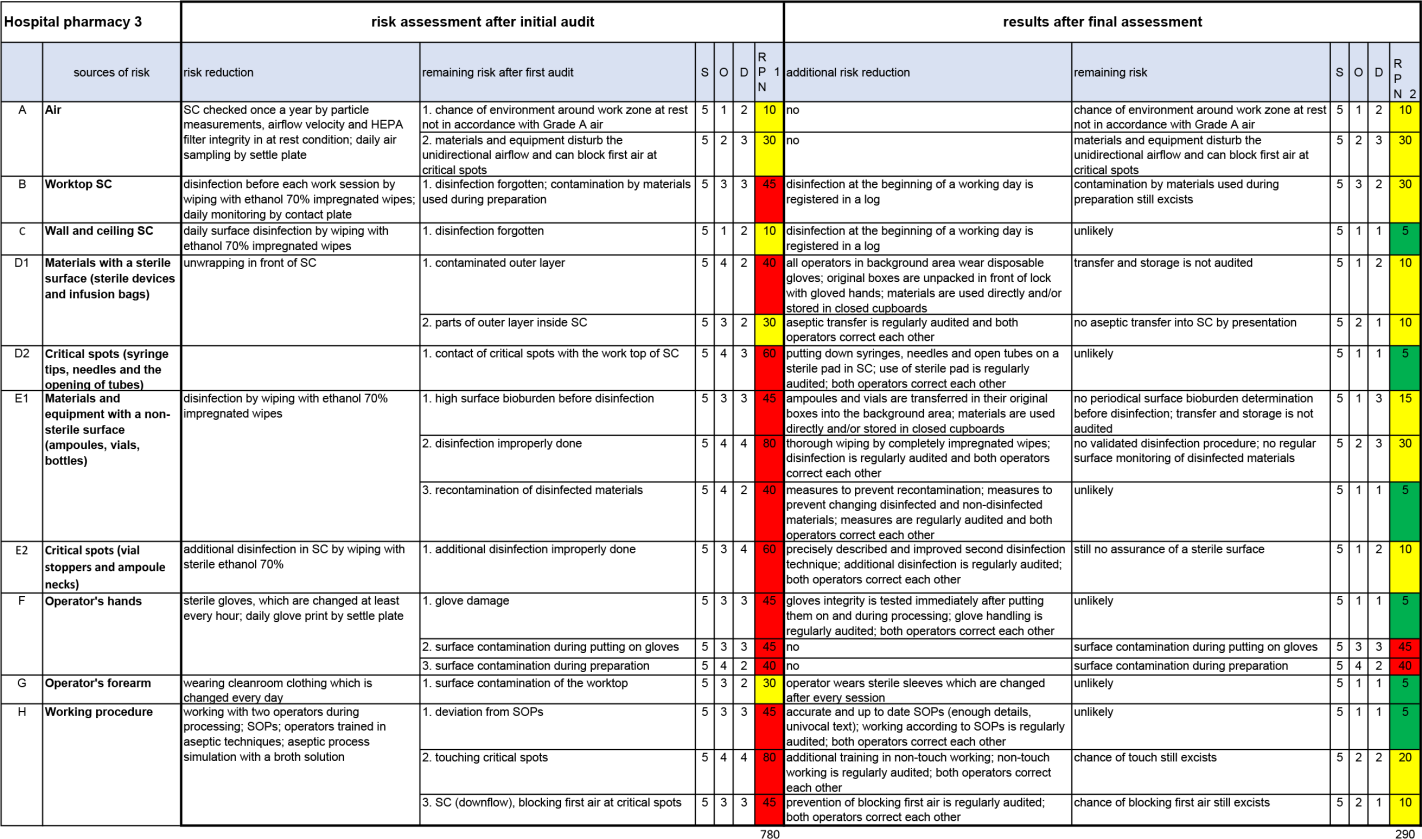
Completed RA and RC template after the final assessment of hospital pharmacy 3. 780, cumulative RPN after the initial audit; 290, cumulative RPN after the final assessment; D, detection; O, occurrence; RPN, risk prioritisation number; S, severity.

**Figure 2:**
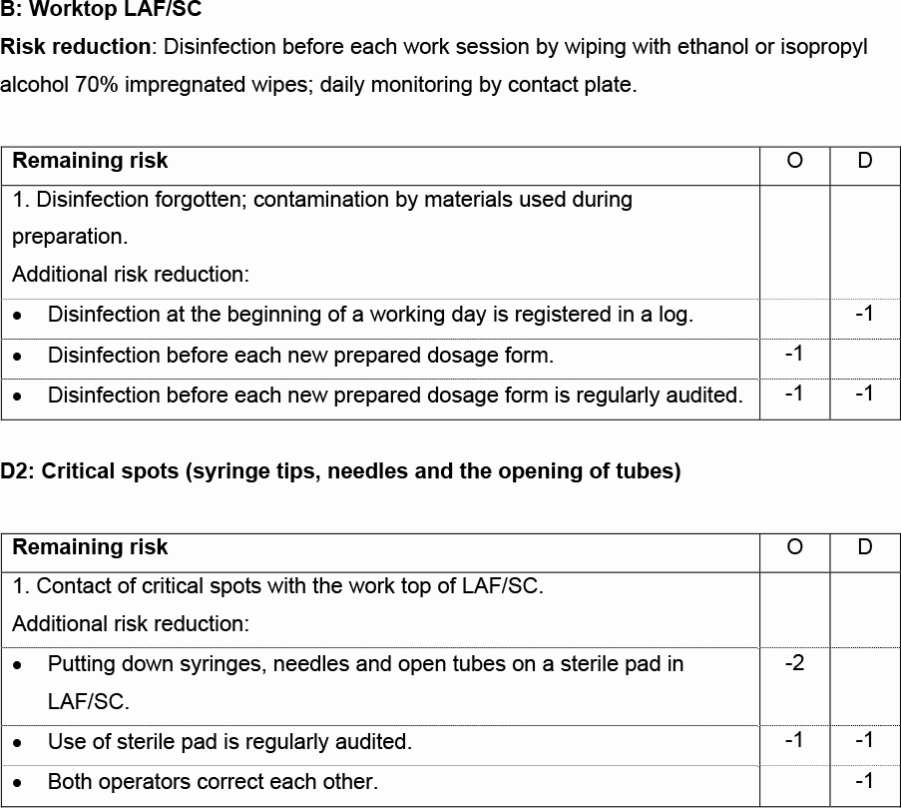
An extract of the checklist with risk reducing measures; the complete checklist is given in online supplemental file 2. Risk reduction and remaining risk, listed in the checklist, were the mean results after the initial audits in the nine participating hospital pharmacies. D, detection; LAF, laminar airflow cabinet; O, occurrence; SC, safety cabinet.

The assessment of aseptic handling in the participating hospital pharmacies consisted of the following steps:

1. At the start of the study each hospital pharmacy was audited by an external pharmaceutical technician and an external hospital pharmacist as described in Part A.^4^ Risk reduction and remaining risks were entered into a risk assessment model, derived from figure 1 of part A. The corresponding RPN values for each source of risk were determined.
2. After this audit, each hospital pharmacy started an improvement programme, using the risk assessment as input. Results from APS and MM were also collected during the study period.
3. The participants were regularly informed about their progress in risk reduction and about the results of APS and MM of each participant.
4. At the end of the study (around 4 years after the start), a final assessment was executed by using a checklist with risk reducing measures for each source of risk. The description of the risk reducing measures was derived from figure 2, 3 and 4 of part B.^5^ The checklist was filled in by the principal investigator (F.A.B.) in consultation with the responsible staff.
5. The remaining risks and corresponding RPN value for each source of risk were determined by using an RA and RC template.

### Microbiological controls

In all participating hospital pharmacies, the standard procedures for APS and MM, described by the Royal Dutch Pharmacists Association, have been used.^7–9^ APS is a broth simulation of the entire process and comprises all critical steps that occur during standard aseptic handling, by withdrawing a solution from a vial or ampoule, dissolving a powder in a vial and adding a solution to an infusion bag or vial. The broth solution used is Tryptone Soya Broth (TSB), Ph Eur. The final product is incubated for 14 days at 30°C and judged on either growth or no growth. The frequency is one APS preparation every working day.

The MM procedures are described in a condensed version in a previous article and consist of passive air sampling by settle plates, glove prints by 90 mm diameter agar plates and worktop prints by contact plates.^8^ The frequency is one sample of each kind of MM every working day.

### Statistics

Contamination recovery rates (CRRs, a definition is given in online supplemental file 1) of MM were compared by p-values using Fisher’s exact test. For calculation of p-values, an online calculator was used.^9^

## Results

### Participating hospital pharmacies

The study started with 10 hospital pharmacies, however in one pharmacy the production of non-hazardous products stopped. Therefore, the results from only nine hospital pharmacies were available for this study.

Table 1 is a short description of the participating hospital pharmacies. Some pharmacies produce a few thousand products each year (mainly parenteral nutrition), some produce more (up to nearly 100 000), for example if batches of syringes are filled or containers for portable infusion pump systems for outpatients are produced.

**Table 1:** Participating hospital pharmacies

Hospital number	Kind of hospital	Year cleanrooms constructed	Facilities and background area	Production 2019 (n)
1	Top clinical	2013	SC in grade C	4300
2	University	2012	SC in grade D	95 600
3	Top clinical	2008	SC in grade D	49 500
4	Top clinical	1995/April 2019	SC in grade C	38 400
5	Top clinical	2013	SC in grade C	15 200
6	University	1981/November 2019	LAF in grade C	13 700
7	Regional	2003	LAF in grade D	3200
8	Regional	2005	LAF in grade C	38 000
9	Regional	1986 /January 2017	LAF and SC in grade D	9800

Kind of hospital: regional, regional hospital; top clinical, large hospital with a level and type of care similar to that offered by university hospitals; university, university hospital. Year cleanrooms constructed: if two dates are given, cleanrooms reconstructed during study period. Facilities and background area: background area, the room in which the LAF/SC is housed; grade C and grade D, EU grade C and grade D environment.^22^ Production 2019 (n): produced number of infusion bags, syringes, containers for portable infusion pump systems in 2019 (all non-hazardous products).

LAF, laminar airflow cabinet; SC, safety cabinet.

### Assessing aseptic handling in nine hospital pharmacies

The risk assessment determined after the initial audit of hospital pharmacy 3 is shown in figure 1 (section ‘risk assessment after initial audit’). The added up RPN value (cumulative RPN) is 780.

The complete checklist, which was used during the final assessment, is given in online supplemental file 2. An extract is given in figure 2. The checklist also gives an instruction for the final assessment.

10.1136/ejhpharm-2021-002747.supp2 Supplementary data

If one of the risk reducing measures given in the checklist was implemented, the value(s) for O and/or D were reduced by the indicated number(s) of risk reduction in online supplemental file 2 and figure 2. For example, ‘Worktop SC’: a log for the registration of the daily disinfection of the worktop was introduced in hospital pharmacy 3 during the study period. This resulted in a reduction of D by one point (see figure 2, B: Worktop LAF/SC). Another example, ‘Critical spots (syringe tips, needles and the opening of tubes)’: all additional risk reductions that were mentioned (see figure 2, D2: Critical spots), were implemented. O reduced by three points and D by two points.

Implemented risk reductions are indicated on the checklist. An example is given in online supplemental file 3 (this is the indicated checklist of hospital pharmacy 3). Additional risk reduction, remaining risk and the new values for O and D are entered into an RA and RC template as shown in figure 1 (section ‘results after final assessment’). The cumulative RPN of hospital pharmacy 3 was reduced to 290 (see figure 1).

10.1136/ejhpharm-2021-002747.supp3 Supplementary data

Table 2 contains the cumulative RPNs of all participating hospital pharmacies after the initial audit and after the final assessment. Table 2 also shows the implemented main additional risk reducing measures and an improvement ratio to express the relative risk reduction of each participant. Online supplemental file 4 contains the completed RA and RC templates of all participating hospital pharmacies.

10.1136/ejhpharm-2021-002747.supp4 Supplementary data

**Table 2:** Cumulative RPN values at the start and at the end of the study, main additional risk reducing measures at the end of the study and improvement ratio

Hospital number	Start	Main additional risk reduction for each source of risk at the end of the study																			End	Improvement ratio (RPN2/RPN1)
	Cumulative RPN (RPN1)	Yearly audit of each operator	Air		Worktop, walls, ceiling		SMD		Ampoules and vials					Hands and forearm of the operator				Working procedures			Cumulative RPN (RPN2)	
			Quarterly non-viable particle counting at rest	Correct position materials	Disinfection registered in a log	Increased frequency worktop disinfection	Wrapped SMD gripped by gloved hands only	Sterile pad	Low surface bioburden before disinfection	Improved disinfection technique	Validated disinfection technique	Monitoring after disinfection	Improved additional disinfection of critical spots	Check of glove damage	Good putting on technique	Glove disinfection before start and every 15 min	Sterile sleeves	Accurate and up to date SOPs	Non-touch working is a major topic during audits	Prevention of blocking first air is a major topic during audits		
1	740	+	–	+	+	+	+	+	+	+	–	–	+	+	+	+	–	–	+	+	230	0.31
2	740	–	–	–	+	–	–	–	–	–	–	–	–	-	+	–	–	–	–	–	700	0.95
3	780	+	–	–	+	–	+	+	+	+	–	–	+	+	–	–	+	+	+	+	290	0.37
4	825	+	+*	–	+	+	+	–	–	+	–	–	–	+†	–	‡	+	–	+	+	365	0.44
5	795	+	–	–	+	+	–	–	+	+	+	–	+	–	+	+	+	+	+	+	280	0.35
6	820	–	–	–	+	+	+	+	+	+	+	–	+	–	+	–	–	+	–	–	505	0.62
7	795	–	–	–	+	–	–	–	–	–	–	–	–	–	–	–	+	–	–	–	725	0.91
8	760	+	–	+	+	+	+	–	+	+	–	+	+	+	+	+	–	+	+	+	280	0.37
9	630	+	–	+	+	+	–	+	–	+	+	+	+	+	+	+	+	+	+	+	235	0.37

+, additional risk reduction implemented; −, additional risk reduction not implemented.

*Continuous particle counting

†Double gloved hands

‡Frequent glove disinfection started at the end of 2019.

End, cumulative RPN after the final assessment; RPN, risk prioritisation number; SMD, sterile medical device; Start, cumulative RPN after the initial audit.

### Microbiological controls

APS results are expressed in contamination rates, which means the percentage of samples with growth. The results are summarised in table 3. Hospital pharmacies 6 and 7 had one sample with growth (2019 and 2016, respectively). The seven other hospital pharmacies had no growth during the study period.

**Table 3:** Results of aseptic process simulation (APS) in nine hospital pharmacies

Hospital number	2016		2017		2018		2019	
	n	CR (%)	n	CR (%)	n	CR (%)	n	CR (%)
1	115	0	117	0	95	0	195	0
2	421	0	309	0	355	0	313	0
3	391	0	414	0	445	0	458	0
4	216	0	227	0	242	0	310	0
5	91	0	93	0	131	0	136	0
6	945	0	987	0	1015	**0.1**	955	0
7	195	**0.51**	160	0	246	0	169	0
8	x	x	96	0	501	0	461	0
9	277	0	384	0	284	0	310	0

Results with growth appear in bold.

CR, contamination rate; hospital number, hospital pharmacy number in this study; n, number of samples examined; x, data not available.

MM results, expressed as CRR, are given in tables 4 and 5. They are derived from the LAF/SC most used in each hospital pharmacy. CRR results above 10% (the limit used in the Netherlands) appear in bold.^10^ To calculate reliable values for CRR, only results of 100 or more samples a year are given in tables 4 and 5.^8^

**Table 4:** Results of air sampling and worktop prints in nine hospital pharmacies

Hospital number	CRR air (%)				CRR worktop (%)			
	2016	2017	2018	2019	2016	2017	2018	2019
1	1.15	1.60	0.27	1.17	2.34	0.80	2,61	0.88
2	5.56	7.19	3.35	5.19	3.59	4.10	4.51	3.29
3	2.14	0.63	1.14	2.03	x	x	x	x
4	4.94	2.94	6.47	1.95	2.72	1.68	1.32	2.62
5	1.38	3.19	3.36	0.88	x	x	6.36	5.67
6	5.84	7.53	5.85	2.73	x	x	x	2.55
7	0.76	0.67	0.52	3.35	x	x	x	3.13
8	3.94	4.73	3.00	2.40	x	x	x	x
9	0.00	0.34	0.81	0.78	3.99	1.01	0.00	1.57

CRR, contamination recovery rate; hospital number, hospital pharmacy number in this study; x, data not available or not enough data available (<100) for calculating a reliable CRR.

**Table 5:** Results of glove prints in nine hospital pharmacies

Hospital number	2016				2017				2018				2019				
	n	pos	neg	CRR (%)	n	pos	neg	CRR (%)	n	pos	neg	CRR (%)	n	pos	neg	CRR (%)	p value
1	390	5	385	1.28	376	13	363	3.46	361	8	353	2.22	349	5	344	1.43	1
2	215	12	203	5.58	146	13	133	8.90	244	15	229	6.15	220	24	196	**10.91**	0.0549
3	882	42	840	4.76	790	34	756	4.30	784	24	760	3.06	727	35	692	4.81	1
4	157	23	134	**14.65**	129	21	108	**16.28**	162	27	135	**16.67**	208	35	173	**17.24**	0.6648
5	290	39	251	**13.45**	226	23	203	**10.18**	299	21	278	7.02	452	37	415	8.19	0.0253
6	585	99	486	**16.92**	623	109	514	**17.50**	517	76	441	**14.70**	1231	92	1139	7.47	0.0001
7	132	10	122	7.58	120	16	104	**13.33**	194	8	186	4.12	179	16	163	8.94	0.8362
8	501	64	437	**12.77**	493	56	437	**11.36**	603	48	555	7.96	637	60	577	9.42	0.0843
9	294	5	289	1.70	298	13	285	4.36	246	8	238	3.25	255	13	242	5.10	0.0307

Results above the 10% limit are in bold.

CRR, contamination recovery rate; n, number of samples examined; neg, number of samples without growth; hospital number, hospital pharmacy number in this study; pos, number of samples with one or more cfu; p value, CRR 2019 compared with CRR 2016.

The results of air and worktop sampling did not change substantially during the study period (see table 4). Therefore, further statistical calculations for these results were not performed.

The results of glove prints from the start of the study (2016) and the end of the study (2019) were compared by Fisher’s exact test. The results of hospital pharmacies 5 and 6 show a statistically significant improvement. The results of hospital pharmacy 9 also show a statistically significant difference. However, 2016 must be considered as a year with extremely low results (see table 5; before 2016, CRRs were 5.76% and 5.71% in 2014 and 2015, respectively).

## Discussion

### Participating hospital pharmacies

There is no correlation between the results (cumulative RPNs as well as microbiological controls) and the kind of hospital, nor between the results and the age of the cleanrooms. Hospital pharmacies 1 and 9 had the overall best results at the end of the study (see tables 2 and 5).

### Comments on the risk reduction of the different sources of risk

In this section comments and additional information about risk reduction of the different sources of risk are given.

#### Air

Most hospital pharmacies own a particle counter, but only one did quarterly non-viable particle counting at rest around the work zone (‘at rest’ and ‘work zone’: definitions are given in online supplemental file 1); this lack of counting is a shame because non-viable particle counting is a simple experiment, while the results will give valuable information about complying with the at rest criteria for airborne particles.^5^

In hospital pharmacies 1, 8 and 9, videos about the risk of blocking first air by materials were used to find the correct position of materials inside LAF/SC.^11^

The results of viable air sampling are already far below the MM limits of up to 10% at the start of the study and did not really change during the study period (see table 4). This is not surprising because there are no distinct sources to contaminate the air inside LAF/SC.^5^

#### Worktop, walls and ceiling of LAF/SC

In all hospital pharmacies, except numbers 2, 3 and 7, the frequency of worktop disinfection increased (see table 2). However, the expected decrease of the CRRs of the worktop prints could not be assessed because the number of samples was often too low to get reliable CRR values (marked as ‘x’ in table 4).^8^ But a positive outcome was the number of pharmacies where daily monitoring of the worktop was being implemented at the end of the study (increased from four to seven hospitals; see table 4).

#### Materials with a sterile surface (sterile medical devices)

Even after thorough disinfection, the worktop has to be considered as a non-sterile surface. Therefore, a sterile pad is advised to prevent contact between critical spots (syringe tips, needles, openings of tubes) and the surface of the worktop.^12^ By the end of the study this pad was being used in four hospital pharmacies (see table 2). An alternative is to put syringes and needles on a sterile holder. Online supplemental file 5 gives an example.

10.1136/ejhpharm-2021-002747.supp5 Supplementary data

#### Materials with a non-sterile surface (ampoules and vials)

Hospital pharmacies 5, 6 and 9 implemented the validated two-towel disinfection technique by using commercially available impregnated sterile polypropylene wipes.^13^ The two-towel technique was also introduced in hospital pharmacies 1 and 8, but these hospital pharmacies used cotton gauzes or medical non-woven wipes, submerged in alcohol 70%. Compared with the commercially available polypropylene wipes, these gauzes and wipes are less expensive. However, a disadvantage of cotton or medical non-woven is the higher emission of particles and fibers. Hospital pharmacies 3 and 4 also improved the disinfection technique (see online supplemental file 4).

Dragging microorganisms across materials with a non-sterile surface is a serious risk.^4^ Therefore, regular surface monitoring after disinfection is strongly advised.^13–15^ This has been implemented in hospital pharmacies 8 and 9 (see table 2). A procedure for routine monitoring of materials with a non-sterile surface is described by Boom and colleagues.^16^

#### Operator’s hands

This section refers to the hands of the primary operator (a definition of which is given in online supplemental file 1). The MM results of glove prints improved during the study period (see table 4). In addition, the frequent glove disinfection which started at the end of 2019 in hospital pharmacy 4 also led to results below the limit of 10% in the next year. Better results for glove prints are not only the result of more frequent glove disinfection, but also the result of more frequent worktop disinfection and better disinfection of materials with a non-sterile surface.^5^ However, if all these improvements are not implemented, a result below the MM limit of up to 10% is also possible (see figure 2, hospital pharmacy 7). Possible explanations for this finding are a low surface bioburden of materials and/or concurrent disinfection of the gloves by the impregnated wipes used during the disinfection of materials and/or frequent glove changes. Besides, the technique of performing glove prints itself can have a great influence on the results.^8 17^ Contact time that is too short, for instance, as well as a too small printed surface of the distal phalanx of the fingers, will have a negative influence on the recovery and therefore on the results.

#### Operator’s forearm

This section refers to the forearm of the primary operator. At the end of the study sterile sleeves were used in five hospital pharmacies. As mentioned in part B, sterile long-sleeved gloves will give the same protection as separate sterile gloves and sleeves.^5^

#### Working procedure

During the whole study period all contamination rates after APS are very low (see table 3). These results show, despite possibilities for risk reduction, that the operators were capable of producing products with a low chance of microbial contamination. However, a few remarks about these results can be made. First, during aseptic handling sometimes the preparation time is longer, and the number of preparation steps is larger compared with the usual applied broth simulation. Therefore, APS is not always a worst case simulation. Second, a more precise way of working during APS, compared with ‘normal’ aseptic handling, is not inconceivable. Third, not all aspects of the way of working can be measured by APS.^18^

In this connection, we emphasise the importance of a yearly audit of all operators as well as stimulating a policy of correcting each other. This not only has a great influence on risk reduction of working procedures, but also on many other sources of risk (see checklist in online supplemental file 2). At the end of the study auditing has been implemented in six hospital pharmacies (see table 2). More information about auditing can be found in part B.^5^

As mentioned in part A, two operators working together during processing is strongly recommended^4^; it makes a policy of correcting each other more workable as well as dividing activities that occur outside and inside LAF/SC and transferring materials into LAF/SC. All hospital pharmacies, except numbers 6, 8 and 9, were already working with two operators during processing at the start of the study. This did not change during the study.

### Reducing the risk of non-sterility in nine hospital pharmacies

The cumulative RPN values at the start of the study varied from 630 to 825 (table 2). At the end of the study the differences were much greater, which leads to a cumulative RPN variation of 230 to 725 (see table 2). The improvement ratios also show great differences (see table 2). A sense of urgency and the time available for the implementation of the additional risk reducing measures are the main reasons for these differences. To enforce process changes, involvement of the responsible staff and the operators is an essential precondition. To enhance this, some hospital pharmacies work with a lean board and stand-up sessions and/or stimulate a policy to correct each other during operation. Additionally, for observing follow-up activities, it is important to use a system for corrective and preventive actions.^19^

Microbiological controls are an important part of the overall assurance of product quality.^20^ Unfortunately, except for glove prints, we did not find a correlation between process improvement (lower cumulative RPN) and the results of microbiological controls. Explanations are given in the subsections above.

It is well known that microbiological controls alone will not cover all sources of risk of non-sterility.^18^ Therefore, according to the principles of a pharmaceutical quality system, it is necessary to evaluate all these sources.^21^ The relevance of each, in combination with the effort to reduce them, can be made clear by the RA and RC model, described in parts A and B.^4 5^

Obviously, the implementation of the risk reducing measures will take time and/or will involve expense. For example, having an audit of two operators requires about 4 hours' work by the auditor.^5^ However, various measures can be implemented by only changing the way of working, without loss of productivity (for example, working without blocking first air on critical spots). Of course, a change itself will take time and energy, but a more robust process is a valuable result. Some measures will even save time, like transfer of ampoules and injection vials in their original white cardboard boxes into the background area (a definition of which is given in online supplemental file 1). This way of working keeps the surface bioburden of ampoules and vials low and shows no measurable influence on the particle burden in the background area.^12^

Application of risk assessment can also cast doubt on habits that have become general practice after years and years. For example, in previous articles we made clear that viable air sampling inside LAF/SC is not sensitive enough for controlling the environment inside LAF/SC or for detecting a filter failure.^5 8^ Therefore, based on the principles of risk assessment, discontinuation of this MM method is a serious option.

### Assessing aseptic handling in other hospital pharmacies

Assessing aseptic handling in other hospital pharmacies can also be done with the checklist available in online supplemental file 2. As mentioned above, an instruction for the assessment is given in the checklist. For determining the RPN values and the remaining risk, a ‘blank’ RA and RC template is available in online supplemental file 6.

10.1136/ejhpharm-2021-002747.supp6 Supplementary data

The determined RPN values can be used for prioritising additional measures for risk reduction. RPN values over 30 are called ‘not safe’ (red) and must be reduced first.^4^

## Conclusion

Systematic and science-based reduction of the risks of non-sterility can be done by using a checklist with risk reducing measures and an RA and RC template. Prospectively, the relevance of each risk reducing measure can be demonstrated by RPN calculations. Of all risk reducing measures, a yearly audit of all operators has the greatest impact on reducing the risk of non-sterility. Microbiological controls are an important part of the overall assurance of product quality. However, a correlation between the results of these controls and the RPN values, looking at the risk of non-sterility, is difficult to prove.

**What this paper adds**

**What is already known on this subject?**

- Aseptic handling has to be executed with aseptic precautions in a laminar airflow cabinet, safety cabinet or isolator.
- The operator is the highest source of risk of non-sterility.

**What does this study adds?**

- Risks of non-sterility and measures to reduce it can be objectified by a risk assessment and risk control model.
- Of all risk reducing measures, a yearly audit of all operators has the greatest impact on reducing the risk of non-sterility.
- The results of microbiological controls are less useful for assessing the risk of non-sterility.

## Data Availability

All data relevant to the study are included in the article or uploaded as supplementary information.
