# Supplementary material for: Reducing the risk of non-sterility of aseptic handling in hospital pharmacies, part C: applying risk assessment and risk control in practice
Source: Eur J Hosp Pharm. 2021 Jul 7;30(3):160–6. doi: 10.1136/ejhpharm-2021-002747 (PMC10176981; doi:10.1136/ejhpharm-2021-002747)
Supplement: Supplementary data [file ejhpharm-2021-002747supp001.pdf]

**SUPPLEMENTARY FILE 1**

## Definitions

- Anteroom is a room inside the clean air area, adjacent to the background area.
- At rest is a room (an environment) complete with all HVAC systems, utilities functioning and with manufacturing equipment installed as specified but without personnel in the facility and the manufacturing equipment is static [1].
- Background area is the room in which the LAF/SC is housed.
- Critical spot is a surface (spot) that may come into contact with a sterile fluid [1]; in the case of aseptic handling a conus of a syringe, a needle, an opening of a tube, an injection puncture, a vial stopper or the neck of an ampoule.
- Contamination recovery rate (CRR) is the percentage of samples that show any microbial recovery, irrespective of the number of cfu [2].
- First air is air from a HEPA filter on a surface without having been obstructed by a non-sterile surface.
- Grade A air is air which is passed through a filter qualified as capable of producing grade A non-viable quality air, but where there is no requirement to continuously perform non-viable monitoring or meet grade A viable monitoring limits [1].
- Primary operator is the operator who performs all tasks inside LAF/SC [3].
- Unidirectional flow is an airflow moving in a single direction, in a robust and uniform manner, and at sufficient speed, to reproducibly sweep particles away from the critical processing or testing area [1].
- Work zone is that part of the worktop inside LAF/SC where the preparation activities are executed.

## References

1. EU Good manufacturing practice (GMP) Annex 1 Revision. Manufacture of sterile medicinal products. December 2017. http://academy.gmp-compliance.org/guidemgr/files/2017_12_PC_ANNEX1_CONSULTATION_DOCUMENT.PDF (accessed 15 January 2021).
2. The United States Pharmacopeia USP 35. The United States Pharmacopeia Convention. Rockville. <1116> Microbiological control and monitoring of aseptic processing environments, 2012.
3. Boom FA, Le Brun PPH, Ris J, Veenbaas TJ, Touw DJ. Reducing the risk on non-sterility of aseptic handling in hospital pharmacies Part A: Risk Assessment. May 2020. Published online by Eu J Hosp Pharm. https://ejhp.bmj.com/content/early/2020/05/08/ejhpharm-2019-002178 (accessed 15 January 2021).
