# Supplementary material for: Reducing the risk of non-sterility of aseptic handling in hospital pharmacies, part C: applying risk assessment and risk control in practice
Source: Eur J Hosp Pharm. 2021 Jul 7;30(3):160–6. doi: 10.1136/ejhpharm-2021-002747 (PMC10176981; doi:10.1136/ejhpharm-2021-002747)
Supplement: Supplementary data [file ejhpharm-2021-002747supp002.pdf]

SUPPLEMENTARY FILE 2

Checklist with risk reducing measures for each source of risk.

This checklist was used during the final assessment of the 9 hospital pharmacies (around 4 years after the start of the study).

In combination with a 'blank' RA & RC template (available in online supplementary file 6) this checklist can also be used for assessing aseptic handling in other hospital pharmacies. It is recommended this is done by two people, all familiar with the SOPs and the way aseptic handling is executed in the assessed hospital pharmacy.
The following steps must be taken:

1. Check for each source of risk if the measures, listed after the words 'Risk reduction', are implemented. If not, implementation is recommended, but should not be taken into account in the tables below.
2. Determine for each remaining risk, listed in the tables below, if it has been carried out or not. If yes, circle the corresponding number(s) in the column 'O' and/or 'D' (see 0nline supplementary file 3 as an example).
3. Add each additional risk reduction and each remaining risk of each source of risk in the blank RA & RC template in the corresponding columns of the section 'results after assessing aseptic handling' (see figure 1 as an example).
4. Diminish the value(s) for O and/or D in the RA & RC template by the corresponding circled numbers in the column 'O' and/or 'D' of the checklist. The new values for O and D have to be entered into the corresponding columns of the section 'results after assessing aseptic handling' (see figure 1 as an example). The new RPNs are calculated automatically.

Checklist, see page 2 - 6

Boom FA, *et al. Eur J Hosp Pharm* 2023; 30:160–166. doi: 10.1136/ejhpharm-2021-002747

hospital pharmacy:	date of the assessment:

Risk reduction and **Remaining risk,** listed in the checklist, were the mean results after the initial audits in the nine participating hospital pharmacies.

D, detection; LAF, laminar airflow cabinet; O, occurrence; SC, safety cabinet.

A: Air inside LAF/SC

Risk reduction: LAF/SC checked once or twice a year by particle measurements, airflow velocity and HEPA filter integrity in at rest* condition. Daily monitoring by settle plate.

**Remaining risk**	O	D
1. Chance of environment around work zone* at rest not in accordance with Grade A air*.<br>Additional risk reduction:		
• non-viable particle counting in work zone at rest at least quarterly		- 1
2. Materials and equipment disturb the unidirectional flow* and can block first air* at critical spots*<br>Additional risk reduction:		
• correct position of materials after investigations by airflow visualization in worst case situation		-1
• position of materials is regularly audited and both operators correct each other	-1	-1

* see definitions

B: Worktop LAF/SC

Risk reduction: Disinfection before each work session by wiping with ethanol or isopropyl alcohol 70% impregnated wipes; daily monitoring by contact plate.

**Remaining risk**	O	D
1. Disinfection forgotten; contamination by materials used during preparation.<br>Additional risk reduction:		
• Disinfection at the beginning of a working day is registered in a log.		-1
• Disinfection before each new prepared dosage form.	-1	
• Disinfection before each new prepared dosage form is regularly audited.	-1	-1

Boom FA, *et al. Eur J Hosp Pharm* 2023; 30:160–166. doi: 10.1136/ejhpharm-2021-002747

C: Wall and ceiling LAF/SC
Risk reduction: Daily surface disinfection by wiping with ethanol or isopropyl alcohol 70% impregnated wipes.

**Remaining risk**	O	D
1. Disinfection forgotten.<br>Additional risk reduction:		
• Disinfection at the beginning of a working day is registered in a log.		-1

D1: Materials with a sterile surface (sterile medical devices and infusion bags)
Risk reduction: Unwrapping in front of LAF/SC.

**Remaining risk**	O	D
1. Contaminated outer layer.<br>Additional risk reduction:		
• All operators in background area* (and anteroom*) wear disposable (sterile) gloves.	-1	
• Unpack original boxes in front of the lock with gloved hands, put materials directly into the lock.	-1	
• Use materials directly and/or store materials in closed cupboards.	-1	
• Transfer and storage are audited at least yearly.		-1
2. Parts of outer layer inside LAF/SC.<br>Additional risk reduction:		
• Aseptic transfer into LAF/SC by presentation.	-1	
• Aseptic transfer is regularly audited and both operators correct each other.	-1	-1

* see definitions

D2: Critical spots* (syringe tips, needles and the opening of tubes)

**Remaining risk**	O	D
1. Contact of critical spots with the work top of LAF/SC.<br>Additional risk reduction:		
• Putting down syringes, needles and open tubes on a sterile pad in LAF/SC.	-2	
• Use of sterile pad is regularly audited.	-1	-1
• Both operators correct each other.		-1

* see definitions

Boom FA, *et al. Eur J Hosp Pharm* 2023; 30:160–166. doi: 10.1136/ejhpharm-2021-002747

E1: Materials and equipment with a non-sterile surface (ampoules, vials, bottles)

Risk reduction: Disinfection by wiping with ethanol or isopropyl alcohol 70%.

**Remaining risk**	O	D
1. High surface bioburden before disinfection.<br>Additional risk reduction:		
• Transfer ampoules and vials in their original boxes into the background area*.	-1	
• Store materials not directly used in their original boxes in the background area in closed cupboards.	-1	
• Periodical surface bioburden determination before disinfection.		-1
• Transfer and storage are audited at least yearly.		-1
2. Disinfection improperly done.<br>Additional risk reduction:		
• Thorough wiping by completely impregnated wipes.	-1	
• Disinfection by a validated disinfection procedure.	-1	
• Regular surface monitoring of disinfected materials.		-2
• Disinfection is regularly audited and both operators correct each other.	-1	-1
c. Recontamination of disinfected materials.<br>Additional risk reduction:		
• Measures to prevent recontamination.	-1	
• Measures to prevent changing disinfected and non-disinfected materials.	-1	
• Measures are regularly audited and both operators correct each other.	-1	-1

* see definitions

E2: Critical spots* (vial stoppers and ampoule necks)

Risk reduction: Additional disinfection in LAF/SC by wiping with sterile ethanol or isopropyl alcohol 70%.

**Remaining risk**	O	D
1. Additional disinfection improperly done.<br>Additional risk reduction:		
• Precisely described and improved additional disinfection technique (thorough wiping and > 30 sec waiting time).	-1	
• Additional disinfection is regularly audited.	-1	-1
• Both operators correct each other.		-1

* see definitions

Boom FA, *et al. Eur J Hosp Pharm* 2023; 30:160–166. doi: 10.1136/ejhpharm-2021-002747

F: Operator's hands

Risk reduction: Sterile gloves, which are changed at least every hour; daily glove print by settle plate.

**Remaining risk**	O	D
1. Glove damage.<br>Additional risk reduction:		
• Check gloves integrity immediately after putting them on and during processing.	-1	
• Glove handling is regularly audited.	-1	-1
• Both operators correct each other.		-1
2. Surface contamination during putting on gloves.<br>Additional risk reduction:		
• Good putting on technique.	-1	
• Putting on gloves is regularly audited.	-1	-1
• Both operators correct each other.		-1
3. Surface contamination during preparation.<br>Additional risk reduction:		
• Glove disinfection before start of each new preparation and every 15 min during a long preparation.	-2	
• Glove disinfection is regularly audited and both operators correct each other.	-1	-1

G: Operator's forearm

Risk reduction: Wearing cleanroom clothing which is changed every day.

**Remaining risk**	O	D
1. Surface contamination of the worktop.<br>Additional risk reduction:		
• Operator wears sterile sleeves which must be changed after every session.	-2	-1

H: Working procedure

Risk reduction: Working with two operators during processing; SOPs; operators trained in aseptic techniques; aseptic process simulation with a broth solution.

**Remaining risk**	O	D
1. Deviation from SOPs.<br>Additional risk reduction:		
• Accurate and up to date SOPs (enough details, univocal text).	-1	
• Working according to SOPs is regularly audited.	-1	-1
• Both operators correct each other.		-1
2. Touching critical spots*.<br>Additional risk reduction:		
• Additional training in non-touch working.	-1	
• Non-touch working is regularly audited.	-1	-1
• Both operators correct each other.		-1
3. Blocking first air* at critical spots.<br>Additional risk reduction:		
• Prevention of blocking first air is regularly audited.	-1	-1
• Both operators correct each other.		-1

* see definitions

Boom FA, *et al. Eur J Hosp Pharm* 2023; 30:160–166. doi: 10.1136/ejhpharm-2021-002747
