# Supplementary material for: Reducing the risk of non-sterility of aseptic handling in hospital pharmacies, part C: applying risk assessment and risk control in practice
Source: Eur J Hosp Pharm. 2021 Jul 7;30(3):160–6. doi: 10.1136/ejhpharm-2021-002747 (PMC10176981; doi:10.1136/ejhpharm-2021-002747)
Supplement: Supplementary data [file ejhpharm-2021-002747supp003.pdf]

SUPPLEMENTARY FILE 3

Filled in checklist of Hospital pharmacy 3.

Checklist, see page 2 - 6

Boom FA, *et al. Eur J Hosp Pharm* 2023; 30:160–166. doi: 10.1136/ejhpharm-2021-002747

hospital pharmacy: Number 3	date of the assessment: May 25, 2020

Risk reduction and **Remaining risk,** listed in the checklist, were the mean results after the initial audits in the nine participating hospital pharmacies.

D, detection; LAF, laminar airflow cabinet; O, occurrence; SC, safety cabinet.

A: Air inside LAF/SC

Risk reduction: LAF/SC checked once or twice a year by particle measurements, airflow velocity and HEPA filter integrity in at rest* condition. Daily monitoring by settle plate.

**Remaining risk**	O	D
1. Chance of environment around work zone* at rest not in accordance with Grade A air*. Additional risk reduction:		
• non-viable particle counting in work zone at rest at least quarterly		- 1
2. Materials and equipment disturb the unidirectional flow* and can block first air* at critical spots* Additional risk reduction:		
• correct position of materials after investigations by airflow visualization in worst case situation		-1
• position of materials is regularly audited and both operators correct each other	-1	-1

* see definitions

B: Worktop LAF/SC

Risk reduction: Disinfection before each work session by wiping with ethanol or isopropyl alcohol 70% impregnated wipes; daily monitoring by contact plate.

**Remaining risk**	O	D
1. Disinfection forgotten; contamination by materials used during preparation. Additional risk reduction:		
• Disinfection at the beginning of a working day is registered in a log.		-1 (circled)
• Disinfection before each new prepared dosage form.	-1	
• Disinfection before each new prepared dosage form is regularly audited.	-1	-1

C: Wall and ceiling LAF/SC

Risk reduction: Daily surface disinfection by wiping with ethanol or isopropyl alcohol 70% impregnated wipes.

Remaining risk	O	D
1. Disinfection forgotten.<br>Additional risk reduction:		
• Disinfection at the beginning of a working day is registered in a log.		-1

D1: Materials with a sterile surface (sterile medical devices and infusion bags)

Risk reduction: Unwrapping in front of LAF/SC.

Remaining risk	O	D
1. Contaminated outer layer.<br>Additional risk reduction:		
• All operators in background area* (and anteroom*) wear disposable (sterile) gloves.	-1	
• Unpack original boxes in front of the lock with gloved hands, put materials directly into the lock.	-1	
• Use materials directly and/or store materials in closed cupboards.	-1	
• Transfer and storage are audited at least yearly.		-1
2. Parts of outer layer inside LAF/SC.<br>Additional risk reduction:		
• Aseptic transfer into LAF/SC by presentation.	-1	
• Aseptic transfer is regularly audited and both operators correct each other.	-1	-1

* see definitions

D2: Critical spots* (syringe tips, needles and the opening of tubes)

Remaining risk	O	D
1. Contact of critical spots with the work top of LAF/SC.<br>Additional risk reduction:		
• Putting down syringes, needles and open tubes on a sterile pad in LAF/SC.	-2	
• Use of sterile pad is regularly audited.	-1	-1
• Both operators correct each other.		-1

* see definitions

Boom FA, *et al*. *Eur J Hosp Pharm* 2023; 30:160–166. doi: 10.1136/ejhpharm-2021-002747

E1: Materials and equipment with a non-sterile surface (ampoules, vials, bottles)

Risk reduction: Disinfection by wiping with ethanol or isopropyl alcohol 70%.

Remaining risk	O	D
1. High surface bioburden before disinfection.<br>Additional risk reduction:		
• Transfer ampoules and vials in their original boxes into the background area*.	-1	
• Store materials not directly used in their original boxes in the background area in closed cupboards.	-1	
• Periodical surface bioburden determination before disinfection.		-1
• Transfer and storage are audited at least yearly.		-1
2. Disinfection improperly done.<br>Additional risk reduction:		
• Thorough wiping by completely impregnated wipes.	-1	
• Disinfection by a validated disinfection procedure.	-1	
• Regular surface monitoring of disinfected materials.		-2
• Disinfection is regularly audited and both operators correct each other.	-1	-1
c. Recontamination of disinfected materials.<br>Additional risk reduction:		
• Measures to prevent recontamination.	-1	
• Measures to prevent changing disinfected and non-disinfected materials.	-1	
• Measures are regularly audited and both operators correct each other.	-1	-1

* see definitions

E2: Critical spots* (vial stoppers and ampoule necks)

Risk reduction: Additional disinfection in LAF/SC by wiping with sterile ethanol or isopropyl alcohol 70%.

Remaining risk	O	D
1. Additional disinfection improperly done.<br>Additional risk reduction:		
• Precisely described and improved additional disinfection technique (thorough wiping and > 30 sec waiting time).	-1	
• Additional disinfection is regularly audited.	-1	-1
• Both operators correct each other.		-1

* see definitions

Boom FA, *et al. Eur J Hosp Pharm* 2023; 30:160–166. doi: 10.1136/ejhpharm-2021-002747

F: Operator's hands

Risk reduction: Sterile gloves, which are changed at least every hour; daily glove print by settle plate.

**Remaining risk**	O	D
1. Glove damage.<br>Additional risk reduction:		
• Check gloves integrity immediately after putting them on and during processing.	-1	
• Glove handling is regularly audited.	-1	-1
• Both operators correct each other.		-1
2. Surface contamination during putting on gloves.<br>Additional risk reduction:		
• Good putting on technique.	-1	
• Putting on gloves is regularly audited.	-1	-1
• Both operators correct each other.		-1
3. Surface contamination during preparation.<br>Additional risk reduction:		
• Glove disinfection before start of each new preparation and every 15 min during a long preparation.	-2	
• Glove disinfection is regularly audited and both operators correct each other.	-1	-1

G: Operator's forearm

Risk reduction: Wearing cleanroom clothing which is changed every day.

**Remaining risk**	O	D
1. Surface contamination of the worktop.<br>Additional risk reduction:		
• Operator wears sterile sleeves which must be changed after every session.	-2	-1

H: Working procedure

Risk reduction: Working with two operators during processing; SOPs; operators trained in aseptic techniques; aseptic process simulation with a broth solution.

**Remaining risk**	O	D
1. Deviation from SOPs.<br>Additional risk reduction:		
• Accurate and up to date SOPs (enough details, univocal text).	-1	
• Working according to SOPs is regularly audited.	-1	-1
• Both operators correct each other.		-1
2. Touching critical spots*.<br>Additional risk reduction:		
• Additional training in non-touch working.	-1	
• Non-touch working is regularly audited.	-1	-1
• Both operators correct each other.		-1
3. Blocking first air* at critical spots.<br>Additional risk reduction:		
• Prevention of blocking first air is regularly audited.	-1	-1
• Both operators correct each other.		-1

* see definitions

Boom FA, *et al. Eur J Hosp Pharm* 2023; 30:160–166. doi: 10.1136/ejhpharm-2021-002747
