# Supplementary material for: Reducing the risk of non-sterility of aseptic handling in hospital pharmacies, part C: applying risk assessment and risk control in practice
Source: Eur J Hosp Pharm. 2021 Jul 7;30(3):160–6. doi: 10.1136/ejhpharm-2021-002747 (PMC10176981; doi:10.1136/ejhpharm-2021-002747)
Supplement: Supplementary data [file ejhpharm-2021-002747supp004.pdf]

Supplementary file 4

Filled in RA&RC templates after the final assessment of the nine participating hospital pharmacies

Hospital pharmacy 1		risk assessment after initial audit						results after final assessment					
	sources of risk	risk reduction	remaining risk after first audit	S	O	D	RPN 1	additional risk reduction	remaining risk	S	O	D	RPN 2
A	**Air**	SC checked once a year by particle measurements, airflow velocity and HEPA filter integrity in at rest condition; daily air sampling by settle plate	1. chance of environment around work zone at rest not in accordance with Grade A air	5	1	2	10	no	chance of environment around work zone at rest not in accordance with Grade A air	5	1	2	10
			2. materials and equipment disturb the unidirectional airflow and can block first air at critical spots	5	2	3	30	correct position of materials after investigation by airflow visualization in worst case situation; position of materials is regularly audited; both operators correct each other	unlikely	5	1	1	5
B	**Worktop SC**	disinfection before each work session by wiping with ethanol 70% impregnated wipes; daily monitoring by contact plate; disinfection at the beginning of a working day is registered in a log	1. contamination by materials used during preparation	5	3	2	30	disinfection before each new prepared dosage form; disinfection before each new prepared dosage form is regularly audited	unlikely	5	1	1	5
C	**Wall and ceiling SC**	daily surface disinfection by wiping with ethanol 70% impregnated wipes; disinfection at the beginning of a working day is registered in a log	unlikely	5	1	1	5	no	unlikely	5	1	1	5
D1	**Materials with a sterile surface (sterile devices and infusion bags)**	unwrapping in front of SC; all operators in background area wear disposable gloves; materials are used directly and/or store in closed cupboards	1. contaminated outer layer	5	2	2	20	original boxes are unpacked in front of lock with gloved hands	transfer and storage is not audited	5	1	2	10
			2. parts of outer layer inside SC	5	3	2	30	aseptic transfer is regularly audited and both operators correct each other	no aseptic transfer into SC by presentation	5	2	1	10
D2	**Critical spots (syringe tips, needles and the opening of tubes)***		1. contact of critical spots with the work top of SC	5	4	3	60	putting down syringes, needles and open tubes on a sterile pad in SC; use of sterile pad is regularly audited; both operators correct each other	unlikely	5	1	1	5
E1	**Materials and equipment with a non-sterile surface (ampoules, vials, bottles)**	disinfection by wiping with ethanol 70% impregnated wipes	1. high surface bioburden before disinfection	5	3	3	45	ampoules and vials are transferred into the anteroom in their original boxes; materials are used directly and/or stored in closed cupboards	no periodical surface bioburden determination before disinfection; transfer and storage is not audited	5	1	3	15
			2. disinfection improperly done	5	4	4	80	thorough wiping by completely impregnated wipes; disinfection is regularly audited and both operators correct each other	no validated disinfection procedure; no regular surface monitoring of disinfected materials	5	2	3	30
			3. recontamination of disinfected materials	5	4	2	40	measures to prevent recontamination; measures to prevent changing disinfected and non-disinfected materials; measures are regularly audited and both operators correct each other	unlikely	5	1	1	5
E2	**Critical spots (vial stoppers and ampoule necks)**	additional disinfection in SC by wiping with sterile ethanol 70%	1. additional disinfection improperly done	5	3	4	60	precisely described and improved additinal disinfection technique; additional disinfection is regularly audited; both operators correct each other	still no assurance of a sterile surface	5	1	2	10
F	**Operator's hands**	sterile gloves, which are changed at least every hour; daily glove print by settle plate	1. glove damage	5	3	3	45	gloves integrity is tested immediately after putting them on and during processing; glove handling is regularly audited; both operators correct each other	unlikely	5	1	1	5
			2. surface contamination during putting on gloves	5	3	3	45	good putting on technique; putting on gloves is regularly audited; both operators correct each other	unlikely	5	1	1	5
			3. surface contamination during preparation	5	4	2	40	glove disinfection before start of each new preparation and every 15 min during a long preparation; glove disinfection is regularly audited and both operators correct each other	unlikely	5	1	1	5
G	**Operator's forearm**	wearing cleanroom clothing which is changed every day	1. surface contamination of the worktop	5	3	2	30	no	surface contamination of the worktop	5	3	2	30
H	**Working procedure**	working with two operators during processing; SOPs; operators trained in aseptic techniques by broth simulations every year; process validation by broth simulation	1. deviation from SOPs	5	3	3	45	no	deviation from SOPs	5	3	3	45
			2. touching critical spots	5	4	4	80	additional training in non-touch working; non-touch working is regularly audited; both operators correct each other	chance of touch still exists	5	2	2	20
			3. SC (downflow), blocking first air at critical spots	5	3	3	45	prevention of blocking first air is regularly audited; both operators correct each other	chance of blocking first air still exists	5	2	1	10
							740						230

HP 1, Hospital pharmacy 1; S, severity; O, occurrence; D, detection; RPN, Risk Prioritization Number. 740, cumulative RPN after the initial audit; 230, cumulative RPN after the final assessment.

Hospital pharmacy 2		risk assessment after initial audit						results after final assessment					
	sources of risk	risk reduction	remaining risk after first audit	S	O	D	RPN 1	additional risk reduction	remaining risk	S	O	D	RPN 2
A	**Air**	SC checked once a year by particle measurements, airflow velocity and HEPA filter integrity in at rest condition; daily air sampling by settle plate	1. chance of environment around work zone at rest not in accordance with Grade A air	5	1	2	10	no	chance of environment around work zone at rest not in accordance with Grade A air	5	1	2	10
			2. materials and equipment disturb the unidirectional airflow and can block first air at critical spots	5	2	3	30	no	materials and equipment disturb the unidirectional airflow which can result in blocking first air at critical spots	5	2	3	30
B	**Worktop SC**	disinfection before each work session by wiping with ethanol 70% impregnated wipes; daily monitoring by contact plate	1. disinfection forgotten; contamination by materials used during preparation	5	3	3	45	disinfection at the beginning of a working day is registered in a log	contamination by materials used during preparation still exists	5	3	2	30
C	**Wall and ceiling SC**	daily surface disinfection by wiping with ethanol 70% impregnated wipes	1. disinfection forgotten	5	1	2	10	disinfection at the beginning of a working day is registered in a log	unlikely	5	1	1	5
D1	**Materials with a sterile surface (sterile devices and infusion bags)**	unwrapping in front of SC; materials are used directly and/or stored in closed cupboards	1. contaminated outer layer	5	3	2	30	all operators in background area wear disposable gloves	no unpacking original boxes in front of lock with gloved hands; transfer and storage is not audited	5	2	2	20
			2. parts of outer layer inside SC	5	3	2	30	no	parts of outer layer inside SC	5	2	3	30
D2	**Critical spots (syringe tips, needles and the opening of tubes)**		1. contact of critical spots with the work top of SC	5	4	3	60	no	contact of critical spots with the work top of SC	5	4	3	60
E1	**Materials and equipment with a non-sterile surface (ampoules, vials, bottles)**	disinfection by wiping with ethanol 70% impregnated wipes	1. high surface bioburden before disinfection	5	3	3	45	no	high surface bioburden before disinfection	5	3	3	45
			2. disinfection improperly done	5	4	4	80	no	disinfection improperly done	5	4	4	80
			3. recontamination of disinfected materials	5	4	2	40	no	recontamination of disinfected materials	5	4	2	40
E2	**Critical spots (vial stoppers and ampoule necks)**	additional disinfection in SC by wiping with sterile ethanol 70%	1. additional disinfection improperly done	5	3	4	60	no	additional disinfection improperly done	5	3	4	60
F	**Operator's hands**	wearing sterile gloves, which are changed at least every hour; good putting on technique; daily glove print by settle plate	1. glove damage	5	2	3	30	no	glove damage	5	2	3	30
			2. putting on gloves is not audited	5	2	3	30	no	putting on gloves is not audited	5	2	3	30
			3. surface contamination during preparation	5	4	2	40	glove disinfection before start of each new preparation and in between every 30 min	no glove disinfection every 15 min during a long preparation; glove disinfection is not regularly audited and both operators don't correct each other	5	3	2	30
G	**Operator's forearm**	wearing cleanroom clothing which is changed every day	1. surface contamination of the worktop	5	2	3	30	no	surface contamination of the worktop	5	2	3	30
H	**Working procedure**	working with two operators during processing; SOPs; operators trained in aseptic techniques; aseptic process simulation with a broth solution	1. deviation from SOPs	5	3	3	45	no	deviation from SOPs	5	3	3	45
			2. touching critical spots	5	4	4	80	no	touching critical spots	5	4	4	80
			c. SC (downflow), blocking first air at critical spots	5	3	3	45	no	blocking first air at critical spots	5	3	3	45
							740						700

HP 2, Hospital pharmacy 2; S, severity; O, occurrence; D, detection; RPN, Risk Prioritization Number. 740, cumulative RPN after the initial audit; 700, cumulative RPN after the final assessment.

Hospital pharmacy 3		risk assessment after initial audit						results after final assessment					
	sources of risk	risk reduction	remaining risk after first audit	S	O	D	RPN 1	additional risk reduction	remaining risk	S	O	D	RPN 2
A	**Air**	SC checked once a year by particle measurements, airflow velocity and HEPA filter integrity in at rest condition; daily air sampling by settle plate	1. chance of environment around work zone at rest not in accordance with Grade A air	5	1	2	10	no	chance of environment around work zone at rest not in accordance with Grade A air	5	1	2	10
			2. materials and equipment disturb the unidirectional airflow and can block first air at critical spots	5	2	3	30	no	materials and equipment disturb the unidirectional airflow and can block first air at critical spots	5	2	3	30
B	**Worktop SC**	disinfection before each work session by wiping with ethanol 70% impregnated wipes; daily monitoring by contact plate	1. disinfection forgotten; contamination by materials used during preparation	5	3	3	45	disinfection at the beginning of a working day is registered in a log	contamination by materials used during preparation still excists	5	3	2	30
C	**Wall and ceiling SC**	daily surface disinfection by wiping with ethanol 70% impregnated wipes	1. disinfection forgotten	5	1	2	10	disinfection at the beginning of a working day is registered in a log	unlikely	5	1	1	5
D1	**Materials with a sterile surface (sterile devices and infusion bags)**	unwrapping in front of SC	1. contaminated outer layer	5	4	2	40	all operators in background area wear disposable gloves; original boxes are unpacked in front of lock with gloved hands; materials are used directly and/or stored in closed cupboards	transfer and storage is not audited	5	1	2	10
			2. parts of outer layer inside SC	5	3	2	30	aseptic transfer is regularly audited and both operators correct each other	no aseptic transfer into SC by presentation	5	2	1	10
D2	**Critical spots (syringe tips, needles and the opening of tubes)**		1. contact of critical spots with the work top of SC	5	4	3	60	putting down syringes, needles and open tubes on a sterile pad in SC; use of sterile pad is regularly audited; both operators correct each other	unlikely	5	1	1	5
E1	**Materials and equipment with a non-sterile surface (ampoules, vials, bottles)**	disinfection by wiping with ethanol 70% impregnated wipes	1. high surface bioburden before disinfection	5	3	3	45	ampoules and vials are transferred in their original boxes into the background area; materials are used directly and/or stored in closed cupboards	no periodical surface bioburden determination before disinfection; transfer and storage is not audited	5	1	3	15
			2. disinfection improperly done	5	4	4	80	thorough wiping by completely impregnated wipes; disinfection is regularly audited and both operators correct each other	no validated disinfection procedure; no regular surface monitoring of disinfected materials	5	2	3	30
			3. recontamination of disinfected materials	5	4	2	40	measures to prevent recontamination; measures to prevent changing disinfected and non-disinfected materials; measures are regularly audited and both operators correct each other	unlikely	5	1	1	5
E2	**Critical spots (vial stoppers and ampoule necks)**	additional disinfection in SC by wiping with sterile ethanol 70%	1. additional disinfection improperly done	5	3	4	60	precisely described and improved additinal disinfection technique; additional disinfection is regularly audited; both operators correct each other	still no assurance of a sterile surface	5	1	2	10
F	**Operator's hands**	sterile gloves, which are changed at least every hour; daily glove print by settle plate	1. glove damage	5	3	3	45	gloves integrity is tested immediately after putting them on and during processing; glove handling is regularly audited; both operators correct each other	unlikely	5	1	1	5
			2. surface contamination during putting on gloves	5	3	3	45	no	surface contamination during putting on gloves	5	3	3	45
			3. surface contamination during preparation	5	4	2	40	no	surface contamination during preparation	5	4	2	40
G	**Operator's forearm**	wearing cleanroom clothing which is changed every day	1. surface contamination of the worktop	5	3	2	30	operator wears sterile sleeves which are changed after every session	unlikely	5	1	1	5
H	**Working procedure**	working with two operators during processing; SOPs; operators trained in aseptic techniques; aseptic process simulation with a broth solution	1. deviation from SOPs	5	3	3	45	accurate and up to date SOPs (enough details, univocal text); working according to SOPs is regularly audited; both operators correct each other	unlikely	5	1	1	5
			2. touching critical spots	5	4	4	80	additional training in non-touch working; non-touch working is regularly audited; both operators correct each other	chance of touch still excists	5	2	2	20
			3. SC (downflow), blocking first air at critical spots	5	3	3	45	prevention of blocking first air is regularly audited; both operators correct each other	chance of blocking first air still excists	5	2	1	10
							780						290

HP 3, Hospital pharmacy 3; S, severity; O, occurrence; D, detection; RPN, Risk Prioritization Number. 780, cumulative RPN after the initial audit; 290, cumulative RPN after the final assessment.

Hospital pharmacy 4		risk assessment after initial audit						results after final assessment					
	sources of risk	risk reduction	remaining risk after first audit	S	O	D	RPN 1	additional risk reduction	remaining risk	S	O	D	RPN 2
A	**Air**	SC checked once a year by particle measurements, airflow velocity and HEPA filter integrity in at rest condition; daily monitoring by settle plate	1. chance of environment around work zone at rest not in accordance with Grade A air	5	1	2	10	continuous particle counting near to work zone	unlikely	5	1	1	5
			2. materials and equipment disturb the unidirectional airflow and can block first air at critical spots	5	2	3	30	no	materials and equipment disturb the unidirectional airflow and can block first air at critical spots	5	2	3	30
B	**Worktop SC**	disinfection before each work session by wiping with ethanol 70% impregnated wipes	1. no daily monitoring by contact plates; disinfection forgotten; contamination by materials used during preparation	5	3	4	60	daily monitoring by contact plates; disinfection at the beginning of a working day is registered in a log; disinfection before each new prepared dosage form is regularly audited	unlikely	5	1	1	5
C	**Wall and ceiling SC**	daily surface disinfection by wiping with ethanol 70% impregnated wipes	1. disinfection forgotten	5	1	2	10	disinfection at the beginning of a working day is registered in a log	unlikely	5	1	1	5
D1	**Materials with a sterile surface (sterile devices and infusion bags)**	unwrapping in front of SC; all operators in background area wear disposable gloves	1. contaminated outer layer	5	3	2	30	original boxes are unpacked in front of lock with gloved hands; materials are used directly and/or stored in closed cupboards	transfer and storage is not audited	5	1	2	10
			2. parts of outer layer inside SC	5	3	2	30	aseptic transfer is regularly audited and both operators correct each other	no aseptic transfer into SC by presentation	5	2	1	10
D2	**Critical spots (syringe tips, needles and the opening of tubes)**		1. contact of critical spots with the work top of SC	5	4	3	60	no	contact of critical spots with the work top of SC	5	4	3	60
E1	**Materials and equipment with a non-sterile surface (ampoules, vials, bottles)**	disinfection by spraying with ethanol 70%	1. high surface bioburden before disinfection	5	3	3	45	no	high surface bioburden before disinfection	5	3	3	45
			2. spraying is an inadequate disinfection technique; disinfection improperly done	5	5	4	100	disinfection by wiping; thorough wiping by completely impregnated wipes; disinfection is regularly audited and both operators correct each other	no validated disinfection procedure; no regular surface monitoring of disinfected materials	5	2	3	30
			3. recontamination of disinfected materials	5	4	2	40	measures to prevent recontamination; measures to prevent changing disinfected and non-disinfected materials; measures are regularly audited and both operators correct each other	unlikely	5	1	1	5
E2	**Critical spots (vial stoppers and ampoule necks)**	additional disinfection of vial stopper in SC by wiping with sterile ethanol 70%	1. no additional disinfection of ampoule necks; additional disinfection improperly done	5	4	4	80	additional disinfection of ampoule necks	additional disinfection improperly done	5	3	4	60
**F**	**Operator's hands**	sterile gloves, which are changed at least every hour; daily glove print by settle plate	1. glove damage	5	3	3	45	hands of operator in SC are double gloved	unlikely	5	1	1	5
			2. surface contamination during putting on gloves	5	3	3	45	no	surface contamination during putting on gloves	5	3	3	45
			3. surface contamination during preparation	5	4	2	40	glove disinfection before start of each new preparation and every 15 min during a long preparation; glove disinfection is regularly audited and both operators correct each other	unlikely	5	1	1	5
G	**Operator's forearm**	wearing cleanroom clothing which is changed every day	1. surface contamination of the worktop	5	3	2	30	operator wears sterile sleeves which are changed after every session	unlikely	5	1	1	5
H	**Working procedure**	working with two operators during processing; SOPs; operators trained in aseptic techniques; aseptic process simulation with a broth solution	1. deviation from SOPs	5	3	3	45	working according to SOPs is regularly audited; both operators correct each other	SOPs can be improved (more details, in particular of critical activities)	5	2	1	10
			2. touching critical spots	5	4	4	80	additional training in non-touch working; operators are regularly audited; both operators correct each other	chance of touch still exists	5	2	2	20
			3. SC (downflow), blocking first air at critical spots	5	3	3	45	prevention of blocking first air is regularly audited; both operators correct each other	chance of blocking first air still exists	5	2	1	10
							825						365

HP 4, Hospital pharmacy 4; S, severity; O, occurrence; D, detection; RPN, Risk Prioritization Number. 825, cumulative RPN after the initial audit; 365, cumulative RPN after the final assessment.

**Hospital pharmacy 5**		**risk assessment after initial audit**						**results after final assessment**					
	sources of risk	risk reduction	remaining risk after first audit	S	O	D	RPN 1	additional risk reduction	remaining risk	S	O	D	RPN 2
A	**Air**	SC checked once a year by particle measurements, airflow velocity and HEPA filter integrity in at rest condition; daily air sampling by settle plate	1. chance of environment around work zone at rest not in accordance with Grade A air	5	1	2	10	no	chance of environment around work zone at rest not in accordance with Grade A air	5	1	2	10
			2. materials and equipment disturb the unidirectional airflow and can block first air at critical spots	5	2	3	30	no	materials and equipment disturb the unidirectional airflow and can block first air at critical spots	5	2	3	30
B	**Worktop SC**	disinfection before each work session by wiping with ethanol 70% impregnated wipes	1. no daily monitoring by contact plates; disinfection forgotten; contamination by materials used during preparation	5	3	4	60	daily monitoring by contact plates; disinfection at the beginning of a working day is registered in a log; disinfection before each new prepared dosage form is regularly audited	unlikely	5	1	1	5
C	**Wall and ceiling SC**	daily surface disinfection by wiping with ethanol 70% impregnated wipes	1. disinfection forgotten	5	1	2	10	disinfection at the beginning of a working day is registered in a log	unlikely	5	1	1	5
D1	**Materials with a sterile surface (sterile devices and infusion bags)**	unwrapping in front of SC	1. contaminated outer layer	5	4	2	40	all operators in background area and anteroom wear disposable gloves; materials are used directly and/or store in closed cupboards	no unpacking original boxes in front of lock with gloved hands; transfer and storage is not audited	5	2	2	20
			2. parts of outer layer inside SC	5	3	2	30	aseptic transfer into SC by presentation; aseptic transfer is regularly audited and both operators correct each other	unlikely	5	1	1	5
D2	**Critical spots (syringe tips, needles and the opening of tubes)**		1. contact of critical spots with the work top of SC	5	4	3	60	no	contact of critical spots with the work top of SC	5	4	3	60
E1	**Materials and equipment with a non-sterile surface (ampoules, vials, bottles)**	disinfection by wiping with ethanol 70% impregnated wipes	1. high surface bioburden before disinfection	5	3	3	45	ampoules and vials are transferred into the anteroom in their original boxes; materials are used directly and/or stored in closed cupboards	no periodical surface bioburden determination before disinfection; transfer and storage is not audited	5	1	3	15
			2. disinfection improperly done	5	4	4	80	thorough wiping by a validated disinfection procedure (two towel technique [15]); disinfection is regularly audited and both operators correct each other	no regular surface monitoring of disinfected materials	5	1	3	15
			3. recontamination of disinfected materials	5	4	2	40	measures to prevent recontamination; measures are regularly audited and both operators correct each other	risk of changing disinfected and non-disinfected materials	5	2	1	10
E2	**Critical spots (vial stoppers and ampoule necks)**	additional disinfection in SC by wiping with sterile ethanol 70%	1. additional disinfection improperly done	5	3	4	60	precisely described and improved additinal disinfection technique; additional disinfection is regularly audited; both operators correct each other	still no assurance of a sterile surface	5	1	2	10
F	**Operator's hands**	sterile gloves, which are changed at least every two hours; daily glove print by settle plate	1. glove damage	5	3	3	45	no	glove damage	5	3	3	45
			2. surface contamination during putting on gloves	5	3	3	45	good putting on technique; putting on technique is regularly audited; both operators correct each other	unlikely	5	1	1	5
			3. surface contamination during preparation	5	4	2	40	glove disinfection before start of each new preparation and every 15 min during a long preparation; glove disinfection is regularly audited and both operators	unlikely	5	1	1	5
G	**Operator's forearm**	wearing cleanroom clothing which is changed every day	1. surface contamination of the worktop	5	3	2	30	operator wears sterile sleeves which are changed after every session	unlikely	5	1	1	5
H	**Working procedure**	working with two operators during processing; SOPs; operators trained in aseptic techniques; aseptic process simulation with a broth solution	1. deviation from SOPs	5	3	3	45	accurate and up to date SOPs (enough details, univocal text); working according to SOPs is regularly audited; both operators correct each other	unlikely	5	1	1	5
			2. touching critical spots	5	4	4	80	additional training in non-touch working; non-touch working is regularly audited; both operators correct each other	still a chance of touch	5	2	2	20
			3. SC (downflow), blocking first air at critical spots	5	3	3	45	prevention of blocking first air is regularly audited; both operators correct each other	still a chance of blocking first air	5	2	1	10
							795						280

HP 5, Hospital pharmacy 5; S, severity; O, occurrence; D, detection; RPN, Risk Prioritization Number. 795, cumulative RPN after the initial audit; 280, cumulative RPN after the final assessment.

**Hospital pharmacy 6**		**risk assessment after initial audit**						**results after final assessment**					
	sources of risk	risk reduction	remaining risk after first audit	S	O	D	RPN 1	additional risk reduction	remaining risk	S	O	D	RPN 2
A	**Air**	LAF checked once a year by particle measurements, airflow velocity and HEPA filter integrity in at rest condition; daily air sampling by settle plate	1. chance of environment around work zone at rest not in accordance with Grade A air	5	1	2	10	no	chance of environment around work zone at rest not in accordance with Grade A air	5	1	2	10
			2. materials and equipment disturb the unidirectional airflow and can block first air at critical spots	5	2	3	30	no	materials and equipment disturb the unidirectional airflow and can block first air at critical spots	5	2	3	30
B	**Worktop LAF**	disinfection before each work session by wiping with ethanol 70% impregnated wipes; daily monitoring by contact plate	1. disinfection forgotten; contamination by materials used during preparation	5	3	3	45	disinfection at the beginning of a working day is registered in a log; disinfection before each new prepared dosage form	disinfection before each new prepared dosage form is not audited	5	2	2	20
C	**Wall and ceiling LAF**	daily surface disinfection by wiping with ethanol 70% impregnated wipes	1. disinfection forgotten	5	1	2	10	disinfection at the beginning of a working day is registered in a log	unlikely	5	1	1	5
D1	**Materials with a sterile surface (sterile devices and infusion bags)**	unwrapping partly in front of LAF	1. contaminated outer layer	5	4	2	40	all operators in background area wear disposable gloves; original boxes are unpacked in front of lock with gloved hands; materials are used directly and/or stored in closed cupboards	transfer and storage is not audited	5	1	2	10
			2. no second operator during processing; parts of outer layer inside LAF	5	4	2	40	all materials are unwrapped in front of LAF	no transfer into LAF by presentation; aseptic transfer is not audited and operators don't correct each other	5	3	2	30
D2	**Critical spots (syringe tips, needles and the opening of tubes)**		1. contact of critical spots with the work top of LAF	5	4	3	60	syringes, needles and open tubes are put down on a sterile pad in LAF	use of a sterile pad is not audited; operators don't correct each other	5	2	3	30
E1	**Materials and equipment with a non-sterile surface (ampoules, vials, bottles)**	disinfection by wiping with ethanol 70% impregnated wipes	1. high surface bioburden before disinfection	5	3	3	45	original boxes are unpacked in front of the lock with gloved hands, materials are put directly into the lock; materials are used directly and/or stored in closed cupboards	no periodical surface bioburden determination before disinfection; transfer and storage is not audited	5	1	3	15
			2. disinfection improperly done	5	4	4	80	thorough wiping by a validated disinfection procedure (two towel technique [15])	no regular surface monitoring of disinfected materials; disinfection procedure is not audited and operators don't correct each other	5	2	4	40
			3. recontamination of disinfected materials	5	4	2	40	measures to prevent recontamination; measures to prevent changing disinfected and non-disinfected materials	measures to prevent recontamination and changing are not audited; operators don't correct each other	5	2	2	20
E2	**Critical spots (vial stoppers and ampoule necks)**	additional disinfection in LAF by wiping with ethanol 70%	1. no use of a sterile disinfectant; additional disinfection improperly done	5	4	4	80	use of a sterile disinfectant; precisely described and improved additinal disinfection technique	additional disinfection is not audited; operators don't correct each other	5	2	4	40
F	**Operator's hands**	sterile gloves, daily glove print by settle plate	1. gloves are not changed regularly; glove damage	5	4	3	60	gloves are changed before each new preparation	no check of glove integrity; glove handling is not audited; operators don't correct each other	5	3	3	45
			2. surface contamination during putting on gloves	5	3	3	45	good putting on technique	putting on gloves is not audited; operators don't correct each other	5	2	3	30
			3. gloves are not changed regularly; surface contamination during preparation	5	5	2	50	new gloves before each new preparation	no glove disinfection every 15 min during a long preparation; glove disinfection is not regularly audited and both operators don't correct each	5	3	2	30
G	**Operator's forearm**	wearing cleanroom clothing which is changed every day	1. surface contamination of the worktop	5	3	2	30	no	surface contamination of the worktop	5	3	2	30
H	**Working procedure**	SOPs; operators trained in aseptic techniques; aseptic process simulation with a broth solution	deviation from SOPs; no second operator during processing	5	3	3	45	accurate and up to date SOPs (enough details, univocal text)	working according to SOPs is not audited; operators don't correct each other	5	2	3	30
			touching critical spots; no second operator during processing	5	4	4	80	additional training in non-touch working	non-touch working is not audited; operators don't correct each other	5	3	4	60
			LAF (crossflow), blocking first air at critical spots; no second operator during processing	5	2	3	30	no	LAF (crossflow), blocking first air at critical spots	5	2	3	30
							820						505

HP 6, Hospital pharmacy 6; S, severity; O, occurrence; D, detection; RPN, Risk Prioritization Number. 820, cumulative RPN after the initial audit; 505, cumulative RPN after the final assessment.

**Hospital pharmacy 7**		**risk assessment after initial audit**						**results after final assessment**					
	sources of risk	risk reduction	remaining risk after first audit	S	O	D	RPN 1	additional risk reduction	remaining risk	S	O	D	RPN 2
A	**Air**	SC checked once a year by particle measurements, airflow velocity and HEPA filter integrity in at rest condition; daily air sampling by settle plate	1. chance of environment around work zone at rest not in accordance with Grade A air	5	1	2	10	no	chance of environment around work zone at rest not in accordance with Grade A air	5	1	2	10
			2. materials and equipment disturb the unidirectional airflow and can block first air at critical spots	5	2	3	30	no	materials and equipment disturb the unidirectional airflow and can block first air at critical spots	5	2	3	30
B	**Worktop SC**	disinfection before each work session by wiping with ethanol 70% impregnated wipes; weekly monitoring by contact plate	1. no daily monitoring by contact plates; disinfection forgotten; contamination by materials used during preparation	5	3	4	60	disinfection at the beginning of a working day is registered in a log	daily glove print by settle plate; contamination by materials used during preparation still exists	5	3	3	45
C	**Wall and ceiling SC**	daily surface disinfection by wiping with ethanol 70% impregnated wipes	1. disinfection forgotten	5	1	2	10	disinfection at the beginning of a working day is registered in a log	unlikely	5	1	1	5
D1	**Materials with a sterile surface (sterile devices and infusion bags)**	unwrapping in front of SC	1. contaminated outer layer	5	4	2	40	no	contaminated outer layer	5	4	2	40
			2. parts of outer layer inside SC	5	3	2	30	no	parts of outer layer inside SC	5	3	2	30
D2	**Critical spots (syringe tips, needles and the opening of tubes)**		1. contact of critical spots with the work top of SC	5	4	3	60	no	contact of critical spots with the work top of SC	5	4	3	60
E1	**Materials and equipment with a non-sterile surface (ampoules, vials, bottles)**	disinfection by wiping with ethanol 70% impregnated wipes	1. high surface bioburden before disinfection	5	3	3	45	no	high surface bioburden before disinfection	5	3	3	45
			2. disinfection improperly done	5	4	4	80	no	disinfection improperly done	5	4	4	80
			3. recontamination of disinfected materials	5	4	2	40	no	recontamination of disinfected materials	5	4	2	40
E2	**Critical spots (vial stoppers and ampoule necks)**	additional disinfection in SC by wiping with sterile ethanol 70%	1. additional disinfection improperly done	5	3	4	60	no	additional disinfection improperly done	5	3	4	60
F	**Operator's hands**	sterile gloves, which are changed at least every hour; daily glove print by settle plate	1. glove damage	5	3	3	45	no	glove damage	5	2	3	30
			2. surface contamination during putting on gloves	5	3	3	45	no	surface contamination during putting on gloves	5	2	3	30
			3. surface contamination during preparation	5	4	2	40	no	surface contamination during preparation	5	3	3	45
G	**Operator's forearm**	wearing cleanroom clothing which is changed every day	1. surface contamination of the worktop	5	2	3	30	operator wears a sterile overcoat, which is changed after every session	unlikely	5	1	1	5
H	**Working procedure**	working with two operators during processing; SOPs; operators trained in aseptic techniques; aseptic process simulation with a broth solution	1. deviation from SOPs	5	3	3	45	no	deviation from SOPs	5	3	3	45
			2. touching critical spots	5	4	4	80	no	touching critical spots	5	4	4	80
			3. SC (downflow), blocking first air at critical spots	5	3	3	45	no	blocking first air at critical spots	5	3	3	45
							795						725

HP 7, Hospital pharmacy 7; S, severity; O, occurrence; D, detection; RPN, Risk Prioritization Number. 795, cumulative RPN after the initial audit; 725, cumulative RPN after the final assessment.

**Hospital pharmacy 8**		**risk assessment after initial audit**						**results after final assessment**					
	sources of risk	risk reduction	remaining risk after first audit	S	O	D	RPN 1	additional risk reduction	remaining risk	S	O	D	RPN 2
A	**Air**	LAF checked 2 times a year by particle measurements, airflow velocity and HEPA filter integrity in at rest condition; daily air sampling by settle plate	1. chance of environment around work zone at rest not in accordance with Grade A air	5	1	2	10	no	chance of environment around work zone at rest not in accordance with Grade A air	5	1	2	10
			2. materials and equipment disturb the unidirectional airflow and can block first air at critical spots	5	2	3	30	correct position of materials after investigations by airflow visualization in worst case situation; position of materials is regularly audited and both operators correct each other	unlikely	5	1	1	5
B	**Worktop LAF**	disinfection before each work session by wiping with ethanol 70% impregnated wipes; weekly monitoring by contact plate; disinfection at the beginning of a working day is registered in a log	1. no daily monitoring by contact plates; contamination by materials used during preparation	5	3	3	45	disinfection before each new prepared dosage form; disinfection before each new prepared dosage form is regularly audited	no daily monitoring by contact plates	5	1	2	10
C	**Wall and ceiling LAF**	daily surface disinfection by wiping with ethanol 70% impregnated wipes; disinfection at the beginning of a working day is registered in a log	unlikely	5	1	1	5	no	unlikely	5	1	1	5
D1	**Materials with a sterile surface (sterile devices and infusion bags)**	unwrapping in front of LAF	1. contaminated outer layer	5	4	2	40	all operators in background area and anteroom wear disposable gloves; original boxes are unpacked in front of lock with gloved hands; materials are used directly and/or stored in closed cupboards	transfer and storage is not audited	5	1	2	10
			2. no second operator during processing; parts of outer layer inside LAF	5	3	2	30	no	no second operator during processing; parts of outer layer inside LAF	5	3	2	30
D2	**Critical spots (syringe tips, needles and the opening of tubes)**		1. contact of critical spots with the work top of LAF	5	4	3	60	no	contact of critical spots with the work top of LAF	5	4	3	60
E1	**Materials and equipment with a non-sterile surface (ampoules, vials, bottles)**	disinfection in anteroom by wiping with ethanol 70% impregnated wipes; well controlled transfer process of disinfected materials into background area; measures to prevent recontamination; measures to prevent changing disinfected and non-disinfected materials	1. high surface bioburden before disinfection	5	3	3	45	ampoules and vials are transferred into the anteroom in their original boxes; materials are used directly and/or stored in closed cupboards	no periodical surface bioburden determination before disinfection; transfer and storage is not audited	5	1	3	15
			2. disinfection improperly done	5	4	4	80	Thorough wiping by completely impregnated wipes; regular surface monitoring of disinfected materials; disinfection is regularly audited	no validated disinfection procedure	5	2	1	10
			3. measures to prevent recontamination and changing are not audited	5	2	2	20	measures are regularly audited and both operators correct each other	unlikely	5	1	1	5
E2	**Critical spots (vial stoppers and ampoule necks)**	additional disinfection in LAF by wiping with sterile ethanol 70%	1. no additional disinfection of ampoule necks; additional disinfection improperly done	5	4	4	80	additional disinfection of ampoule necks; precisely described and improved additinal disinfection technique; additional disinfection is regularly audited	still no assurance of a sterile surface; no second operator during processing	5	1	3	15
F	**Operator's hands**	sterile gloves, which are changed at least every hour; daily glove print by settle plate	1. glove damage	5	3	3	45	gloves integrity is tested immediately after putting them on and during processing; glove handling is regularly audited	no second operator during processing; parts of outer layer inside LAF	5	1	2	10
			2. surface contamination during putting on gloves	5	3	3	45	good putting on technique; putting on technique is regularly audited	no second operator during processing; parts of outer layer inside	5	1	2	10
			3. surface contamination during preparation	5	4	2	40	glove disinfection before start of each new preparation and every 15 min during a long preparation; glove disinfection is regularly audited	unlikely	5	1	1	5
G	**Operator's forearm**	wearing cleanroom clothing which is changed every day	1. surface contamination of the worktop	5	3	2	30	no	surface contamination of the worktop	5	2	3	30
H	**Working procedure**	SOPs; operators trained in aseptic techniques; aseptic process simulation with a broth solution	1. deviation from SOPs; no second operator during processing	5	3	3	45	accurate and up to date SOPs (enough details, univocal text); working according to SOPs is regularly audited	no second operator during processing	5	1	2	10
			2. touching critical spots; no second operator during processing	5	4	4	80	additional training in non-touch working; non-touch working is regularly audited	still a chance of touch; no second operator during processing	5	2	3	30
			3. LAF (crossflow), blocking first air at critical spots; no second operator during processing	5	2	3	30	prevention of blocking first air is regularly audited	no second operator during processing	5	1	2	10
							760						280

HP 8, Hospital pharmacy 8; S, severity; O, occurrence; D, detection; RPN, Risk Prioritization Number. 760, cumulative RPN after the initial audit; 280, cumulative RPN after the final assessment.

**Hospital pharmacy 9**		**risk assessment after initial audit**						**results after final assessment**					
	sources of risk	risk reduction	remaining risk after first audit	S	O	D	RPN 1	additional risk reduction	remaining risk	S	O	D	RPN 2
A	**Air**	LAF checked once a year by particle measurements, airflow velocity and HEPA filter integrity in at rest condition; daily air sampling by settle plate	1. chance of environment around work zone at rest not in accordance with Grade A air	5	1	2	10	no	chance of environment around work zone at rest not in accordance with Grade A air	5	1	2	10
			2. materials and equipment disturb the unidirectional airflow and can block first air at critical spots	5	2	3	30	correct position of materials after investigations by airflow visualization in worst case situation; position of materials is regularly audited	unlikely	5	1	1	5
B	**Worktop LAF**	disinfection before each work session by wiping with isopropyl alcohol 70% impregnated wipes; daily monitoring by contact plate; disinfection at the beginning of a working day is registered in a log	1. contamination by materials used during preparation	5	3	2	30	disinfection before each new prepared dosage form; disinfection before each new prepared dosage form is regularly audited	unlikely	5	1	1	5
C	**Wall and ceiling LAF**	daily surface disinfection by wiping with isopropyl alcohol 70% impregnated wipes; disinfection at the beginning of a working day is registered in a log	unlikely	5	1	1	5	no	unlikely	5	1	1	5
D1	**Materials with a sterile surface (sterile devices and infusion bags)**	unwrapping in front of LAF; all operators in background area wear disposable gloves; materials are used directly and/or store in closed cupboards; aseptic transfer is regularly audited	1. contaminated outer layer	5	2	2	20	no	contaminated outer layer	5	2	2	20
			2. no second operator during processing; parts of outer layer inside LAF	5	3	2	30	no	no second operator during processing; parts of outer layer inside LAF	5	3	2	30
D2	**Critical spots (syringe tips, needles and the opening of tubes)**		1. contact of critical spots with the work top of LAF	5	4	3	60	putting down syringes, needles and open tubes on a sterile pad in LAF; use of sterile pad is regularly audited	no second operator during processing	5	1	2	10
E1	**Materials and equipment with a non-sterile surface (ampoules, vials, bottles)**	disinfection by wiping with isopropyl ethanol 70% impregnated wipes; disinfection is regularly audited; measures to prevent changing disinfected and non-disinfected materials which are audited regularly	1. high surface bioburden before disinfection	5	3	3	45	no	high surface bioburden before disinfection	5	3	3	45
			2. disinfection improperly done	5	4	4	80	thorough wiping by a validated disinfection procedure (two towel technique [15]); regular surface monitoring of disinfected materials; disinfection is regularly audited	unlikely	5	1	1	5
			3. recontamination of disinfected materials	5	3	2	30	measures to prevent recontamination; measures are regularly audited	unlikely	5	1	1	5
E2	**Critical spots (vial stoppers and ampoule necks)**	additional disinfection in LAF by wiping with sterile ethanol 70%; additional disinfection is regularly audited	1. additional disinfection can be improved	5	2	3	30	precisely described and improved additinal disinfection technique	still no assurance of a sterile surface; no second operator during processing	5	1	3	15
F	**Operator's hands**	sterile gloves, which are changed at least every hour; daily glove print by settle plate	1. glove damage	5	3	3	45	gloves integrity is tested immediately after putting them on and during processing; glove handling is regularly audited	no second operator during processing	5	1	2	10
			2. surface contamination during putting on gloves	5	3	3	45	good putting on technique; putting on technique is regularly audited	no second operator during processing	5	1	2	10
			3. surface contamination during preparation	5	4	2	40	glove disinfection before start of each new preparation and and every 15 min during a long preparation; glove disinfection is regularly audited	unlikely	5	1	1	5
G	**Operator's forearm**	waring cleanroom clothing which is changed every day	1. surface contamination of the worktop	5	3	2	30	operator wears sterile sleeves which are changed after every session	unlikely	5	1	1	5
H	**Working procedure**	SOPs; operators trained in aseptic techniques; aseptic process simulation with a broth solution; non-touch working and prevention of blocking first air are regularly audited	1. deviation from SOPs; no second operator during processing	5	3	3	45	accurate and up to date SOPs (enough details, univocal text); working according to SOPs is regularly audited	no second operator during processing	5	1	2	10
			2. touching critical spots; no second operator during processing	5	3	3	45	additional training in non-touch working	still a chance of touch; no second operator during processing	5	2	3	30
			3. LAF (crossflow), blocking first air at critical spots; no second operator during processing	5	1	2	10	no	no second operator during processing	5	1	2	10
							630						235

HP 9, Hospital pharmacy 9; S, severity; O, occurrence; D, detection; RPN, Risk Prioritization Number. 630, cumulative RPN after the initial audit; 235, cumulative RPN after the final assessment.
