# Supplementary material for: Reducing the risk of non-sterility of aseptic handling in hospital pharmacies, part C: applying risk assessment and risk control in practice
Source: Eur J Hosp Pharm. 2021 Jul 7;30(3):160–6. doi: 10.1136/ejhpharm-2021-002747 (PMC10176981; doi:10.1136/ejhpharm-2021-002747)

SUPPLEMENTARY FILE 5

Sterile holder for syringes and needles, which can be used inside LAF/SC instead of a sterile pad

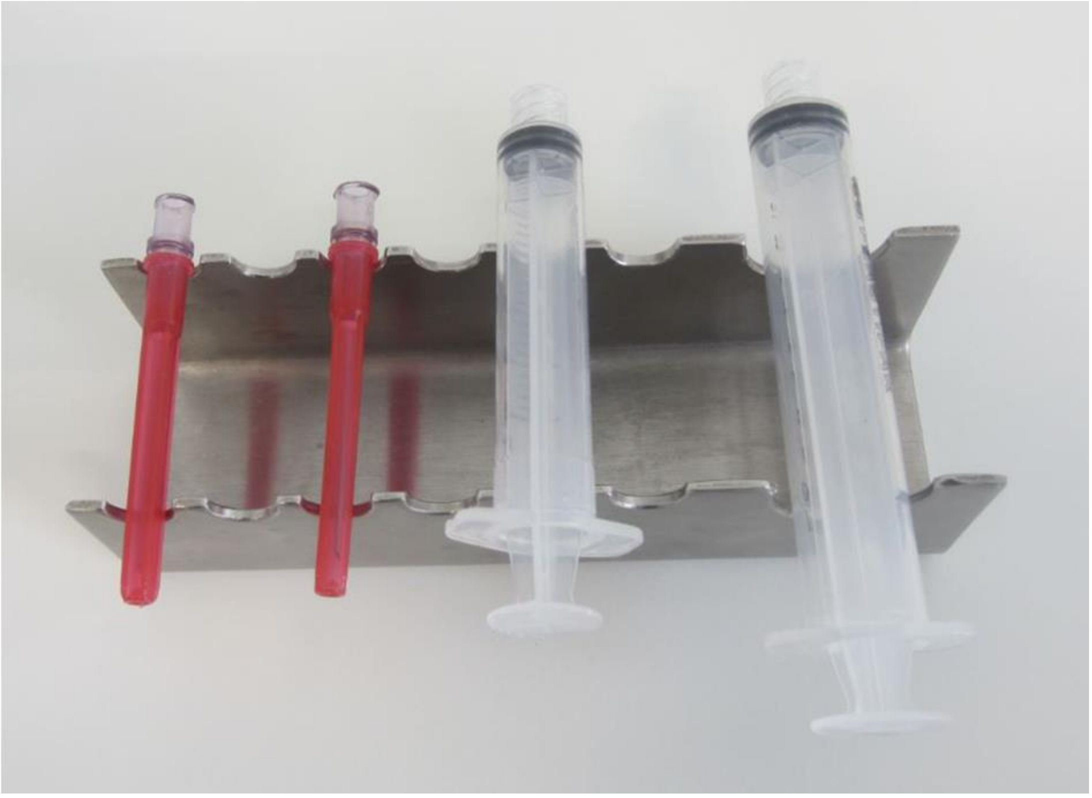

Supplement: Supplementary data [file ejhpharm-2021-002747supp005.pdf]
