# Supplementary material for: Reducing the risk of non-sterility of aseptic handling in hospital pharmacies, part C: applying risk assessment and risk control in practice
Source: Eur J Hosp Pharm. 2021 Jul 7;30(3):160–6. doi: 10.1136/ejhpharm-2021-002747 (PMC10176981; doi:10.1136/ejhpharm-2021-002747)
Supplement: Supplementary data [file ejhpharm-2021-002747supp006.pdf]

Supplementary file 6

'Blank' RA & RC template for determining RPN values and remaining risk,
after assessing aseptic handling by using the checklist of supplementary file 2

								results after assessing aseptic handling					
	sources of risk	general applied risk reduction	remaining risk after general applied risk reduction	S	O	D	RPN 1	additional risk reduction according to checklist II (see online supplementary file 2), used during the assessment	remaining risk after the assessment	S	O	D	RPN 2
A	**Air**	LAF/SC checked once a year by particle measurements, airflow velocity and HEPA filter integrity in at rest condition; daily monitoring by settle plate	1. chance of environment around work zone at rest not in accordance with Grade A air	5	1	2	10						0
			2. materials and equipment disturb the unidirectional airflow and can block first air at critical spots	5	2	3	30						0
B	**Worktop LAF/SC**	disinfection before each work session by wiping with ethanol or isopropyl alcohol 70% impregnated wipes; daily monitoring by contact plate	1. disinfection forgotten; contamination by materials used during preparation	5	3	3	45						0
C	**Wall and ceiling LAF/SC**	daily surface disinfection by wiping with ethanol or isopropyl alcohol 70% impregnated wipes	1. disinfection forgotten	5	1	2	10						0
D1	**Materials with a sterile surface (sterile devices and infusion bags)**	unwrapping in front of LAF/SC	1. contaminated outer layer	5	4	2	40						0
			2. parts of outer layer inside LAF/SC	5	3	2	30						0
D2	**Critical spots (syringe tips, needles and the opening of tubes)**		1. contact of critical spots with the work top of LAF/SC	5	4	3	60						0
E1	**Materials and equipment with a non-sterile surface (ampoules, vials, bottles)**	disinfection by wiping with ethanol or isopropyl alcohol 70%	1. high surface bioburden before disinfection	5	3	3	45						0
			2. disinfection improperly done	5	4	4	80						0
			3. recontamination of disinfected materials	5	4	2	40						0
E2	**Critical spots (vial stoppers and ampoule necks)**	additional disinfection in LAF/SC by wiping with sterile ethanol or isopropyl alcohol 70%	1. additional disinfection improperly done	5	3	4	60						0
F	**Operator's hands**	sterile gloves, which are changed at least every hour; daily monitoring by glove print 5 fingers	1. glove damage	5	3	3	45						0
			2. surface contamination during putting on gloves	5	3	3	45						0
			3. surface contamination during preparation	5	4	2	40						0
G	**Operator's forearm**	wearing cleanroom clothing which is changed every day	1. surface contamination of the worktop	5	3	2	30						0
H	**Working procedure**	working with two operators; SOP; operators trained in aseptic techniques by broth simulations every year; process validation by broth simulation	1. deviation from SOPs	5	3	3	45						0
			2. touching critical spots	5	4	4	80						0
			3a. crossflow: blocking first air at critical spots	5	2	3	30						0
			or 3b. downflow: blocking first air at critical spots	5	3	3	45						0
							765/780						0

'Blank' RA & RC template for determining RPN values and remaining risk, after assessing aseptic handling by using the checklist of online supplementary file 2.

'General applied risk reduction' corresponds to the measures for each source of risk, listed after the words 'Risk reduction' in the checklist; 'Remaining risk after general applied risk reduction' corresponds to the text for each source of risk listed below 'Remaining risk' in the checklist. Both are the mean results after the audits in the nine participating hospital pharmacies.

S, severity; O, occurrence; D, detection; RPN, Risk Prioritization Number. 765/780, cumulative RPN 1 if aseptic handling is done in crossflow (3a) or downflow (3b) respectively.

The Excel version of this template is available and can be ordered by the principal investigator (fritsboom70@gmail.com)
